# Supplementary material for: A broadly generalizable stabilization strategy for sarbecovirus fusion machinery vaccines
Source: bioRxiv. 2023 Dec 20:2023.12.12.571160. Preprint. [Version 2] doi: 10.1101/2023.12.12.571160 (PMC10760017; doi:10.1101/2023.12.12.571160)
Supplement: Supplement 1 [file NIHPP2023.12.12.571160v2-supplement-1.pdf]

bioRxiv preprint doi: https://doi.org/10.1101/2023.12.12.571160; this version posted December 20, 2023. The copyright holder for this preprint (which was not certified by peer review) is the author/funder, who has granted bioRxiv a license to display the preprint in perpetuity. It is made available under aCC-BY 4.0 International license.

Supplementary Table 1. SARS-CoV-2 S_2 prefusion design details with range and mutations.

Design	Residue range	HexaPro	VFLIP inter-protomer disulfide bonds	Intra-protomer disulfide bonds
**C-44**	686-1208	A892P, A899P, A942P, V987P	Y707C-T883C	F970C-G999C
**Design**	**Residue range**	**Additional mutations to theC-44 background**		
**E-31**	686-1208	T961F		
**E-32**	686-1208	D994E		
**E-33**	686-1208	Q1005R		
**E-60**	686-1208	F888P, A893P, N907E, T961F, D994Q, T998Q, Q1010M, Q1011M, I1018Y, N1023M		
**E-69**	686-1208	N907E, T961F, D994Q, Q1011M, I1018Y		
**F-53**	701-1208	N907E, T961F, D994Q, Q1011M, I1018Y		
**SARS1 S_2**	683-1190	N889E, T943F, D976Q, Q993M, I1000Y		
**PRD-0038 S_2**	684-1191	N890E, V940Q, T944F, D977Q, Q994M, I1001Y		

Supplementary Table 2. CryoEM data collection and refinement statistics.

bioRxiv preprint doi: https://doi.org/10.1101/2023.12.12.571160; this version posted December 20, 2023. The copyright holder for this preprint (which was not certified by peer review) is the author/funder, who has granted bioRxiv a license to display the preprint in perpetuity. It is made available under aCC-BY 4.0 International license.

	E-31	E-60	E-69
**Data collection and processing**			
**Magnification**	105,000	105,000	105,000
**Voltage (kV)**	300	300	300
**Electron exposure (e⁻/Å²)**	63	63	63
**Defocus range (μm)**	-0.2 - -7.0	-0.2 - -3.0	-0.1 - -3.27
**Pixel size (Å)**	1	1	0.843
**Symmetry imposed**	C3	C3	C3
**Final particle images (no.)**	671,707	144,044	319,001
**Map resolution (Å)**	2.7	3.5	3.0
**FSC threshold**	0.143	0.143	0.143
**Map sharpening *B*factor (Å²)**	-119.4	-143.9	-127.6
**Validation**			
**MolProbity score**	1.14	1.11	1.0
**Clashscore**	1.29	1.47	1.33
**Poor rotamers (%)**	0	0.53	0.26
**Ramachandran plot**			
**Favored (%)**	95.89	96.55	97.28
**Allowed (%)**	3.65	3.45	2.27
**Disallowed (%)**	0.46	0	0.45

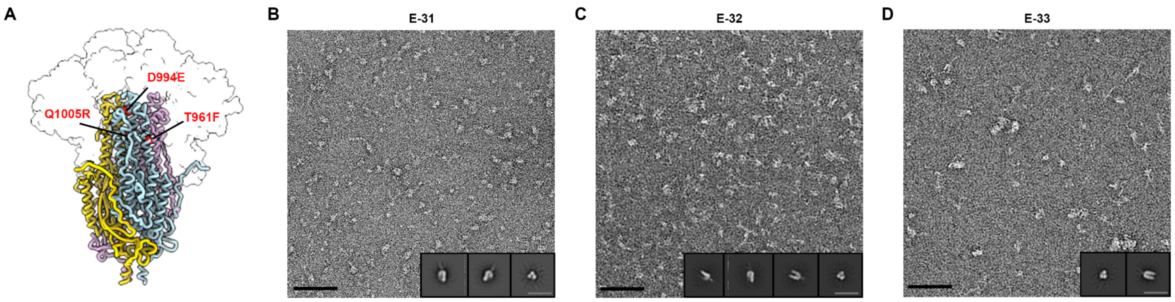

bioRxiv preprint doi: https://doi.org/10.1101/2023.12.12.571160; this version posted December 20, 2023. The copyright holder for this preprint (which was not certified by peer review) is the author/funder, who has granted bioRxiv a license to display the preprint in perpetuity. It is made available under aCC-BY 4.0 International license.

Supplementary Figure 1. Ultrastructural characterization of designed SARS-CoV-2 S_2 prefusion immunogens with single mutations. A, Ribbon diagram of prefusion SARS-CoV-2 S (PDB 6VXX[34]) highlighting all three positions shown in red (T961F, D994E, Q1005R) that were individually mutated to attempt to stabilize the metastable fusion machinery in the prefusion conformation. The S_1 subunit is shown as a transparent surface and glycans are omitted for clarity. . **B-D,** EM analysis of negatively stained E-31 (T961F) (B), E-32 (D994E) (C), and E-33 (Q1005R) (D). Insets: 2D class averages showing compact and splayed open prefusion S_2 trimers. The scale bar represents 50 nm (black) or 200 Å (insets, gray).

bioRxiv preprint doi: https://doi.org/10.1101/2023.12.12.571160; this version posted December 20, 2023. The copyright holder for this preprint (which was not certified by peer review) is the author/funder, who has granted bioRxiv a license to display the preprint in perpetuity. It is made available under aCC-BY 4.0 International license.

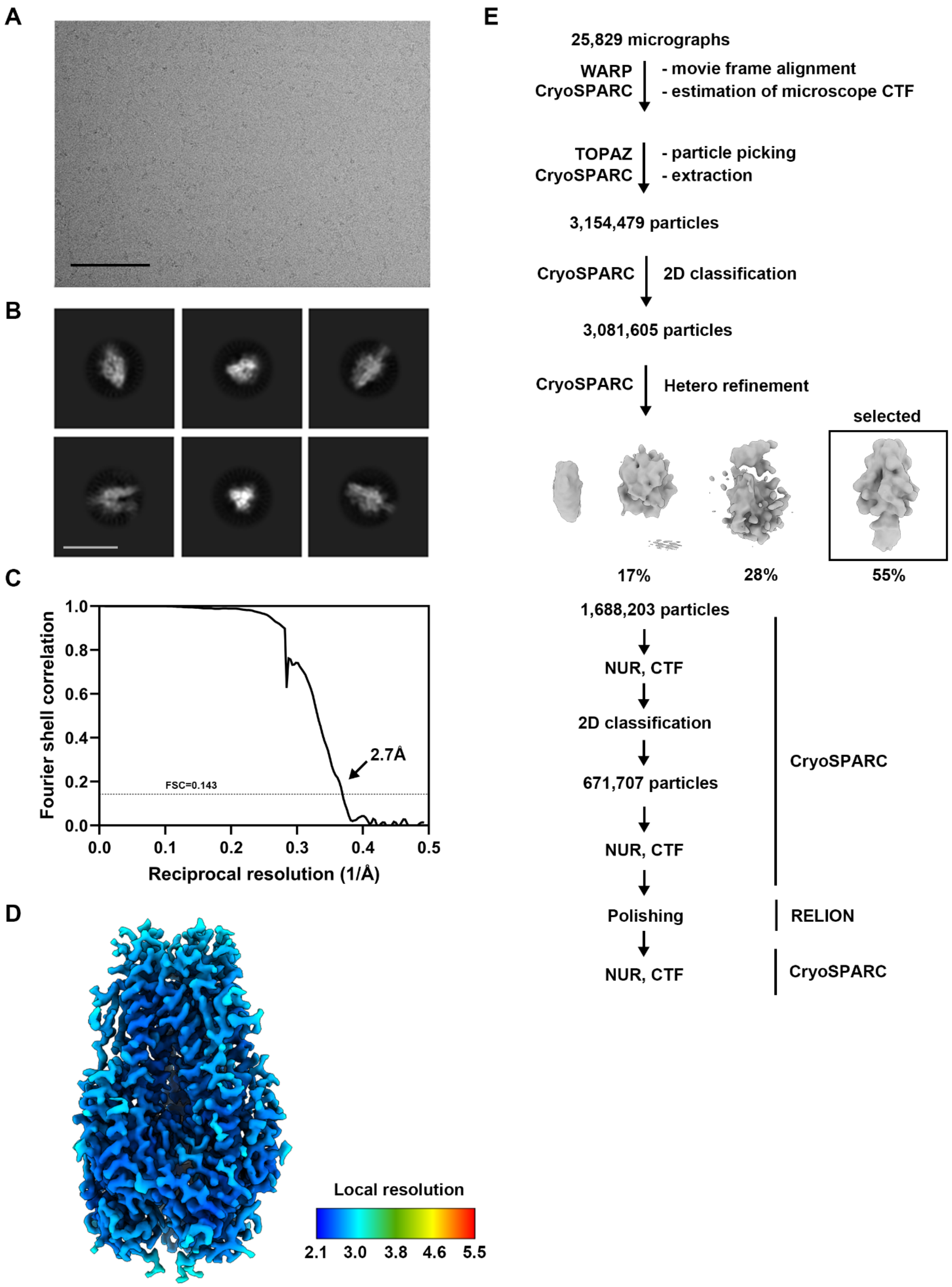

Supplementary Figure 2. CryoEM data collection and refinement of SARS-CoV-2 S_2 E-31.

bioRxiv preprint doi: https://doi.org/10.1101/2023.12.12.571160; this version posted December 20, 2023. The copyright holder for this preprint (which was not certified by peer review) is the author/funder, who has granted bioRxiv a license to display the preprint in perpetuity. It is made available under aCC-BY 4.0 International license.

A, B, Representative electron micrograph (A) and 2D class averages (B) of SARS-CoV-2 S_2 E-31 embedded in vitreous ice. The scale bar represents 100 nm (A) or 160Å (B). **C,** Gold-standard Fourier shell correlation curve for the cryoEM reconstruction. The 0.143 cutoff is indicated with a gray dashed line. **D,** SARS-CoV-2 S_2 E-31 cryoEM map colored by local resolution as determined using cryoSPARC. **E,** Data processing flowchart. NUR, CTF: non-uniform refinement with per-particle defocus refinement.

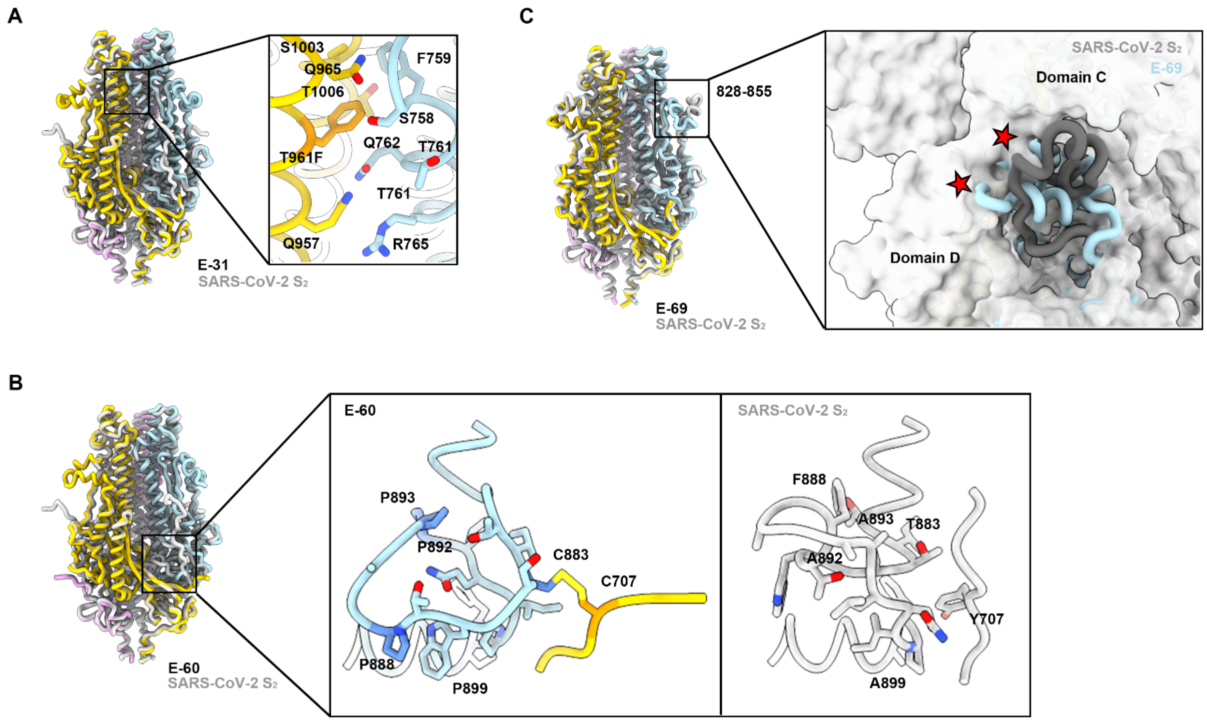

Supplementary Figure 3. Structural details of prefusion stabilized S_2 subunit designs. A, E-31 superimposed to SARS-CoV-2 S_2 (6VXX, gray). Zoomed in view of T961F mutation and proximal residues (inset). B, E-60 superimposed to SARS-CoV-2 S_2 (6VXX, gray). Zoomed in

bioRxiv preprint doi: https://doi.org/10.1101/2023.12.12.571160; this version posted December 20, 2023. The copyright holder for this preprint (which was not certified by peer review) is the author/funder, who has granted bioRxiv a license to display the preprint in perpetuity. It is made available under aCC-BY 4.0 International license.

view of residues 875-906 of E-60 (inset, left) and SARS2 S_2 (6VXX, gray) (inset, right). Mutated residues are shown in blue and orange. **C**, E-69 superimposed to SARS-CoV-2 S (PDB 6XR8). Steric clash shown with red stars.

bioRxiv preprint doi: https://doi.org/10.1101/2023.12.12.571160; this version posted December 20, 2023. The copyright holder for this preprint (which was not certified by peer review) is the author/funder, who has granted bioRxiv a license to display the preprint in perpetuity. It is made available under aCC-BY 4.0 International license.

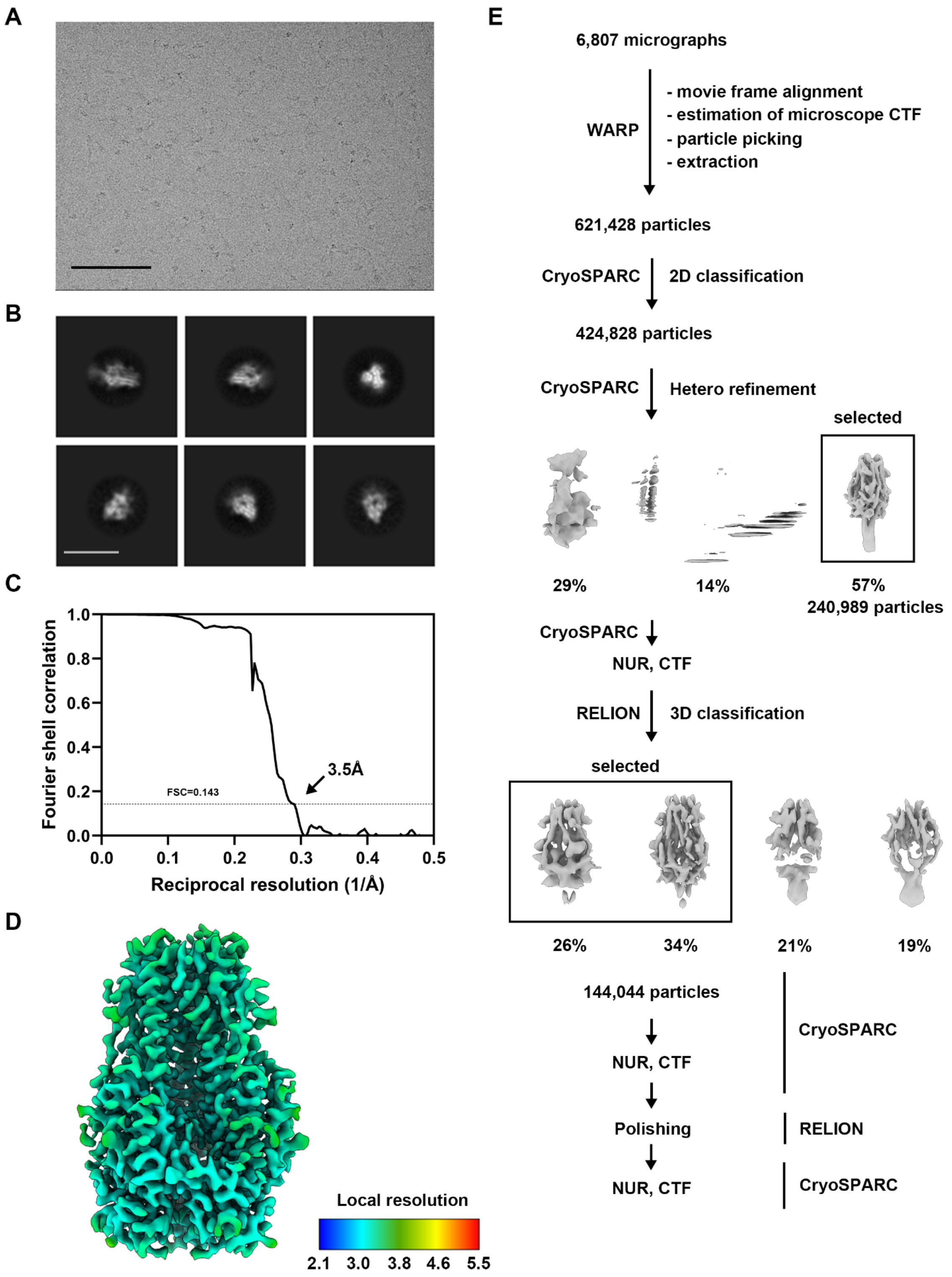

Supplementary Figure 4. CryoEM data collection and refinement of SARS-CoV-2 S_2 E-60.

bioRxiv preprint doi: https://doi.org/10.1101/2023.12.12.571160; this version posted December 20, 2023. The copyright holder for this preprint (which was not certified by peer review) is the author/funder, who has granted bioRxiv a license to display the preprint in perpetuity. It is made available under aCC-BY 4.0 International license.

A, B, Representative electron micrograph (A) and 2D class averages (B) of SARS-CoV-2 S_2 E-60 embedded in vitreous ice. The scale bar represents 100 nm (A) or 160Å (B). **C,** Gold-standard Fourier shell correlation curve for the cryoEM reconstruction. The 0.143 cutoff is indicated with a gray dashed line. **D,** SARS-CoV-2 S_2 E-60 cryoEM map colored by local resolution as determined using cryoSPARC. **E,** Data processing flowchart. NUR, CTF: non-uniform refinement with per-particle defocus refinement.

bioRxiv preprint doi: https://doi.org/10.1101/2023.12.12.571160; this version posted December 20, 2023. The copyright holder for this preprint (which was not certified by peer review) is the author/funder, who has granted bioRxiv a license to display the preprint in perpetuity. It is made available under aCC-BY 4.0 International license.

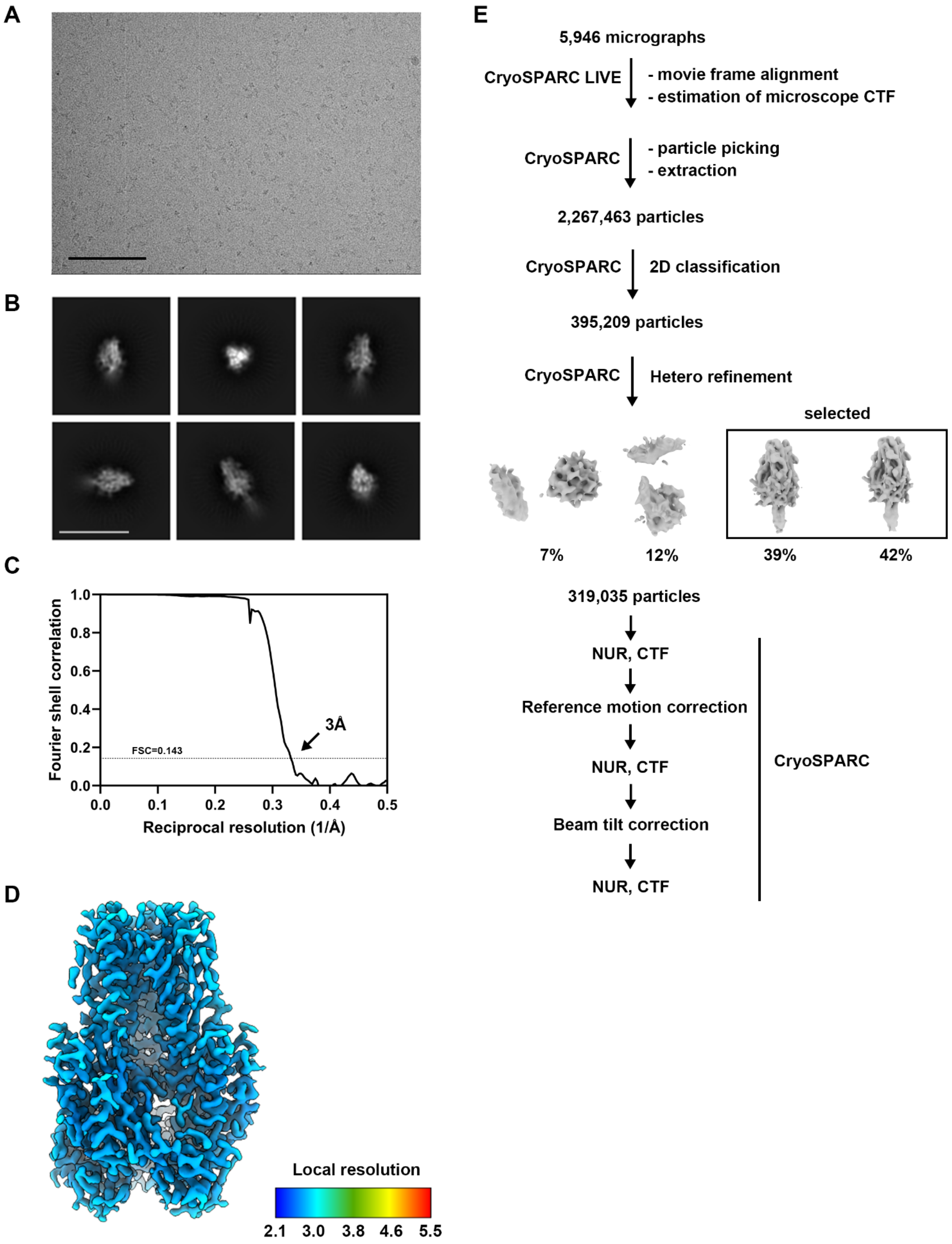

Supplementary Figure 5. CryoEM data collection and refinement of SARS-CoV-2 S_2 E-69.

bioRxiv preprint doi: https://doi.org/10.1101/2023.12.12.571160; this version posted December 20, 2023. The copyright holder for this preprint (which was not certified by peer review) is the author/funder, who has granted bioRxiv a license to display the preprint in perpetuity. It is made available under aCC-BY 4.0 International license.

A, B, Representative electron micrograph (A) and 2D class averages (B) of SARS-CoV-2 S_2 (E-69) embedded in vitreous ice. The scale bar represents 100 nm (A) or 200Å (B). **C,** Gold-standard Fourier shell correlation curve for the cryoEM reconstruction. The 0.143 cutoff is indicated with a gray dashed line. **D,** SARS-CoV-2 S_2 (E-69) colored by local resolution as determined using cryoSPARC. **E,** Data processing flowchart. NUR, CTF: non-uniform refinement with per-particle defocus refinement.

bioRxiv preprint doi: https://doi.org/10.1101/2023.12.12.571160; this version posted December 20, 2023. The copyright holder for this preprint (which was not certified by peer review) is the author/funder, who has granted bioRxiv a license to display the preprint in perpetuity. It is made available under aCC-BY 4.0 International license.

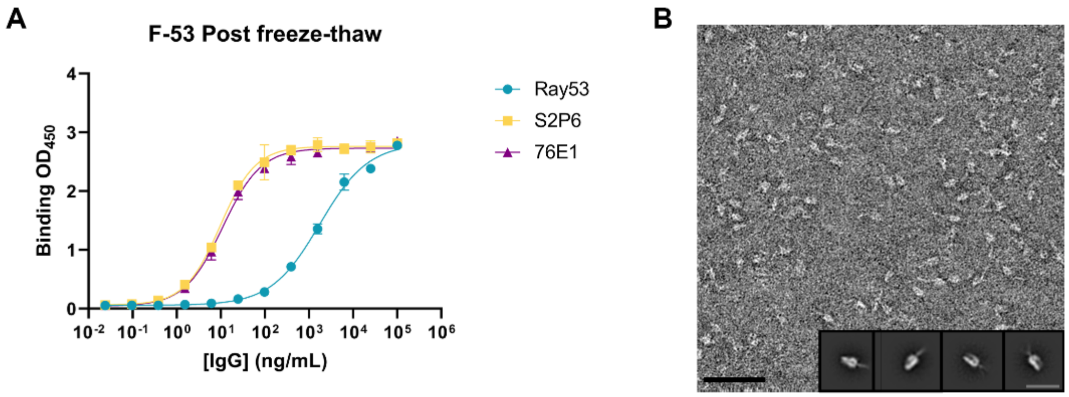

Supplementary Figure 6. Ultrastructural characterization of SARS-CoV-2 S_2 prefusion design F-53. A, Evaluation of binding of a panel of monoclonal antibodies to SARS-CoV-2 S_2 E-69 by ELISA. **B,** EM analysis of negatively stained purified F-53. Insets: 2D class averages showing compact/splayed open prefusion S_2 trimers. The scale bar represents 50 nm (black) or 200 Å (insets, gray).

bioRxiv preprint doi: https://doi.org/10.1101/2023.12.12.571160; this version posted December 20, 2023. The copyright holder for this preprint (which was not certified by peer review) is the author/funder, who has granted bioRxiv a license to display the preprint in perpetuity. It is made available under aCC-BY 4.0 International license.

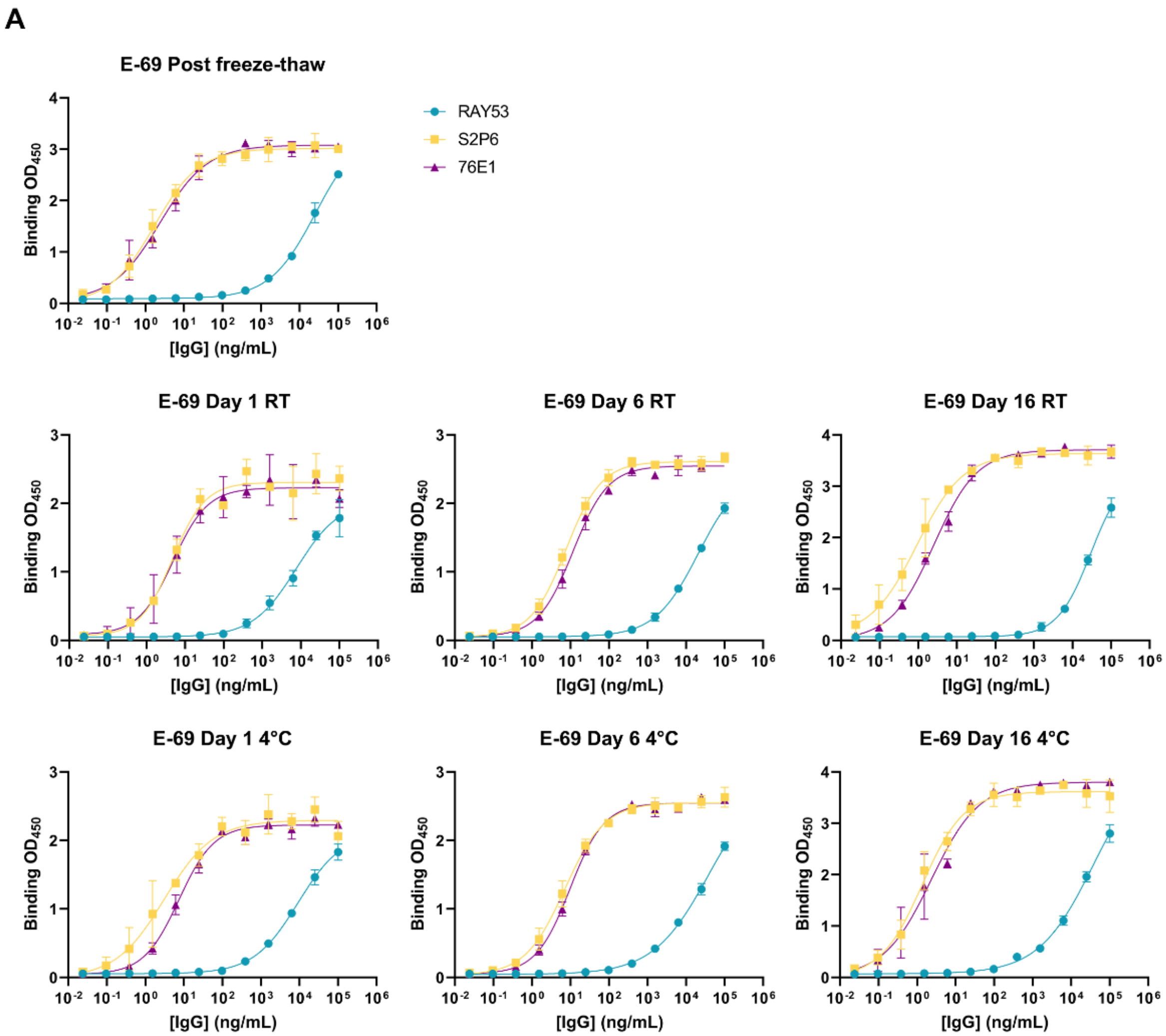

Supplementary Figure 7. Retention of antigenicity of SARS-CoV-2 S_2 E-69. A, Evaluation of binding of a panel of monoclonal antibodies to SARS-CoV-2 S_2 E-69 under various storage conditions measured by ELISA.

bioRxiv preprint doi: https://doi.org/10.1101/2023.12.12.571160; this version posted December 20, 2023. The copyright holder for this preprint (which was not certified by peer review) is the author/funder, who has granted bioRxiv a license to display the preprint in perpetuity. It is made available under aCC-BY 4.0 International license.

A

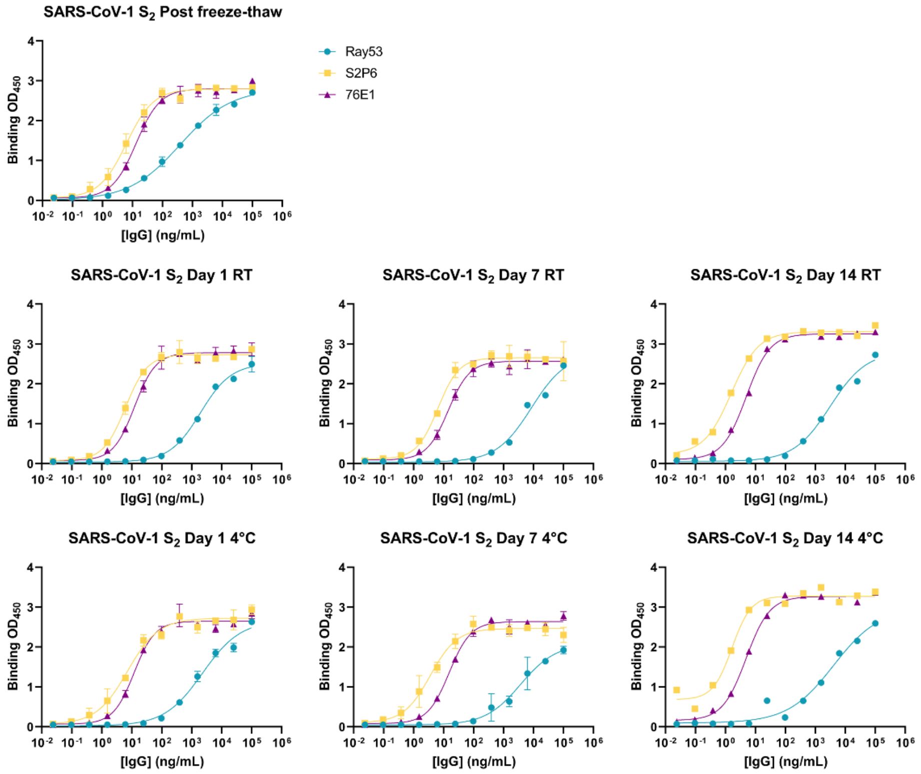

B

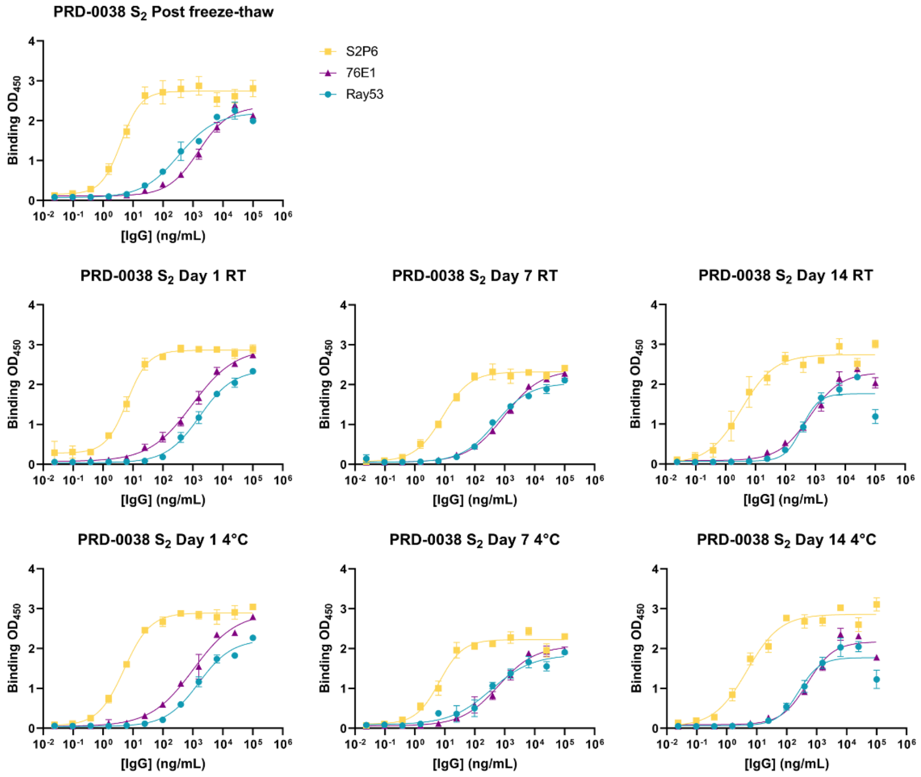

bioRxiv preprint doi: https://doi.org/10.1101/2023.12.12.571160; this version posted December 20, 2023. The copyright holder for this preprint (which was not certified by peer review) is the author/funder, who has granted bioRxiv a license to display the preprint in perpetuity. It is made available under aCC-BY 4.0 International license.

Supplementary Figure 8. Retention of antigenicity of SARS-CoV-2 S_2 E-69. A,B, Evaluation of binding of a panel of monoclonal antibodies to SARS-CoV-1 S_2 (A) and PRD-0038 S_2 (B) under various storage conditions measured by ELISA.

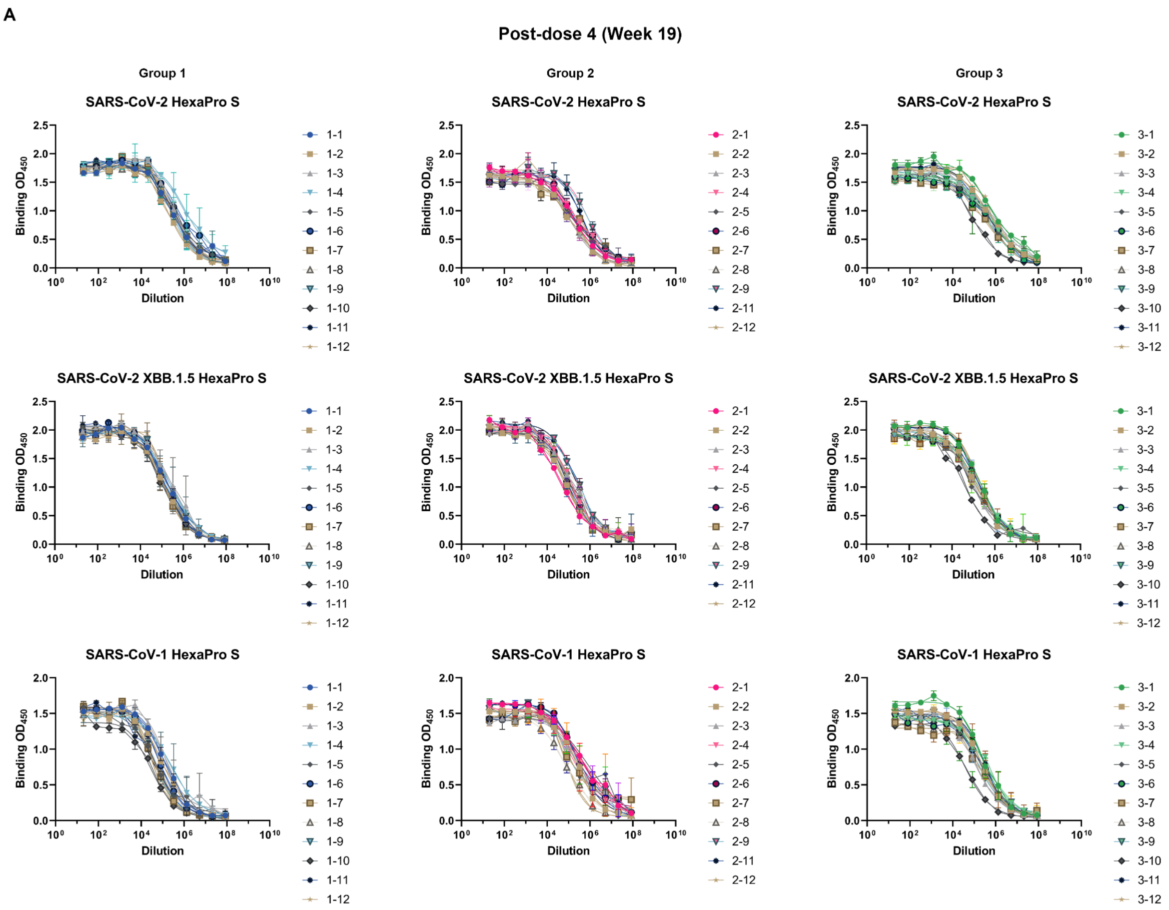

Supplementary Figure 9. Analysis of vaccine-elicited serum antibody binding titers against various S trimers by ELISA. A, Representative dose-response curves of serum antibody binding to SARS-CoV-2 Hexapro S, XBB.1.5 Hexapro S, and SARS-CoV-1 Hexapro S using sera obtained 2 weeks post dose 4. Each dot represents two technical replicates. The assay has been repeated twice and the representative graph is shown.

bioRxiv preprint doi: https://doi.org/10.1101/2023.12.12.571160; this version posted December 20, 2023. The copyright holder for this preprint (which was not certified by peer review) is the author/funder, who has granted bioRxiv a license to display the preprint in perpetuity. It is made available under aCC-BY 4.0 International license.

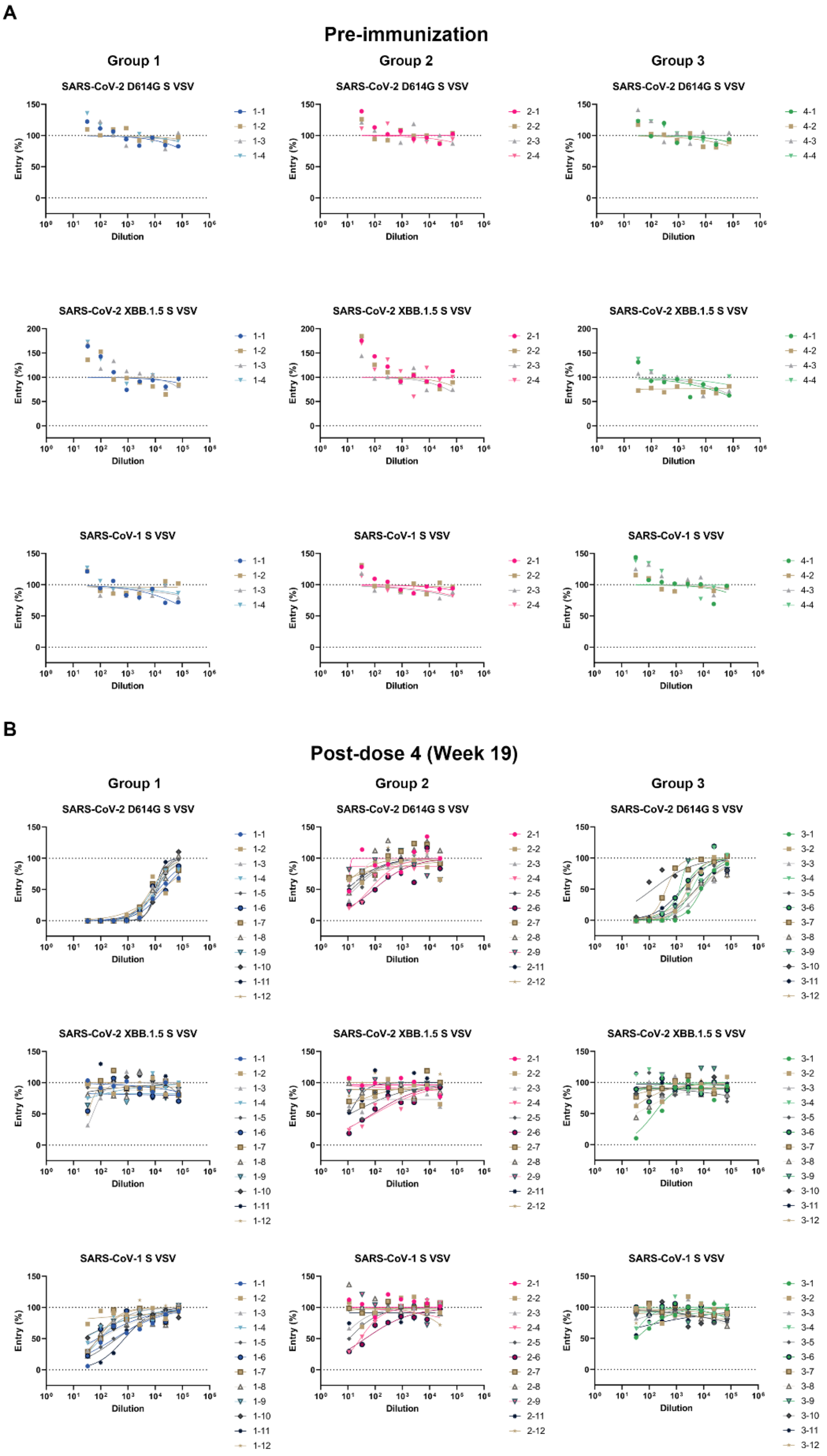

bioRxiv preprint doi: https://doi.org/10.1101/2023.12.12.571160; this version posted December 20, 2023. The copyright holder for this preprint (which was not certified by peer review) is the author/funder, who has granted bioRxiv a license to display the preprint in perpetuity. It is made available under aCC-BY 4.0 International license.

Supplementary Figure 10. Analysis of vaccine-elicited serum neutralizing antibody titers. **A,B,** Dose-response curves of serum neutralizing antibody titers against the SARS-CoV-2 Wu/G614, XBB.1.5 and SARS-CoV-1 S VSV pseudotypes using sera obtained prior to immunization (A) and two weeks post dose 4 (B), as indicated by the color key. Representative curves from three biological replicates are shown.
